# Group 3 Pulmonary Hypertension: Mechanistic Insights, Clinical Challenges, and Evolving Therapies

**DOI:** 10.3390/jcm15176810

**Published:** 2026-09-02

**Authors:** Steven C. Liu, Madeline Ku, Priyanka Mohnani, Tanvi Borse, Bharat Bajantri

**Affiliations:** 1 Department of Internal Medicine, Parkview Health, 11109 Parkview Plaza Dr., Fort Wayne, IN 46845, USA; madeline.ku@parkview.com (M.K.); priyanka.mohnani@parkview.com (P.M.); tanviborse1998@gmail.com (T.B.); 2 Department of Pulmonary Critical Care Medicine, Parkview Health, 11109 Parkview Plaza Dr., Fort Wayne, IN 46845, USA; bharat.bajantri@parkview.com

**Keywords:** Group 3 pulmonary hypertension, pulmonary vascular remodeling, pulmonary hypertension associated with interstitial lung disease, chronic obstructive pulmonary disease-associated pulmonary hypertension

## Abstract

Group 3 pulmonary hypertension is a common and clinically significant complication of chronic lung disease and hypoxia that is associated with impaired functional capacity, right ventricular dysfunction, and increased mortality. Historically viewed as a consequence of underlying parenchymal lung disease, Group 3 pulmonary hypertension is now recognized as a complex disorder involving pulmonary vascular remodeling, dysregulated molecular signaling, and maladaptive cardiopulmonary interactions. Advances in translational research have improved our understanding of the mechanisms driving disease progression and have informed the development of targeted therapeutic strategies. This review provides an overview of the current understanding of Group 3 pulmonary hypertension, including its pathophysiology, diagnostic evaluation, and evolving treatment landscape. Specifically, this review dives into the reason behind limited therapeutic success, the outcomes of previous clinical trials, and the emergence of lung-selective approaches that seek to balance pulmonary vascular benefit with preservation of gas exchange. Collectively, these developments highlight both the progress made and the ongoing need for improved phenotyping and novel therapeutic approaches in the Group 3 pulmonary hypertension patient population.

## 1. Introduction

Pulmonary hypertension (PH) is a complex hemodynamic disorder characterized by elevated pressure in the pulmonary circulation and may arise from a wide range of cardiovascular and respiratory diseases. Because PH encompasses diverse underlying mechanisms and clinical presentations, its diagnosis and management require a multidisciplinary approach. Advances in clinical research, imaging modalities, and hemodynamic assessment have significantly improved the understanding of PH, leading to updated international guidelines that emphasize early detection, accurate classification, and individualized management strategies [1]. Among the clinical subgroups, pulmonary hypertension associated with chronic lung disease and hypoxia (Group 3 PH) is one of the most common forms, frequently occurring in patients with chronic obstructive pulmonary disease (COPD), interstitial lung disease (ILD), and other hypoxia-related disorders. Patients commonly present with exertional dyspnea, reduced exercise tolerance, fatigue, and progressive hypoxemia, although these manifestations often overlap with symptoms of the underlying lung disease. The development of PH in this setting is multifactorial and is associated with reduced functional capacity, increased oxygen requirements, and worse clinical outcomes. Diagnosis remains challenging due to overlapping symptoms with the underlying pulmonary disease, often requiring integrated diagnostic evaluation [1,2]. Therapeutic strategies have traditionally focused on optimizing the underlying pulmonary disease and correcting hypoxemia. However, the role of targeted pulmonary vasodilator therapies has remained controversial due to concerns for worsening ventilation–perfusion (V/Q) mismatch and inconsistent results in earlier studies. More recently, increasing research efforts have led to clinical trials investigating therapies targeting nitric oxide, endothelin, and prostacyclin pathways [1,2,3]. In this review, we examine the current clinical trial evidence evaluating targeted therapies in Group 3 pulmonary hypertension and their implications for management.

## 2. Background

PH is hemodynamically defined as a mean pulmonary arterial pressure (mPAP) > 20 mmHg at rest, with precapillary PH characterized by pulmonary vascular resistance (PVR) > 2 Wood units and pulmonary capillary wedge pressure ≤ 15 mmHg. Group 3 PH occurs in association with chronic lung diseases and/or hypoxia and is typically precapillary in nature. Severe disease has been proposed when PVR exceeds 5 Wood units, identifying a subgroup with worse prognosis [1,4].

PH affects approximately 1% of the global population, rising to nearly 10% in individuals over 65 years of age. Group 3 PH represents the second most common cause after left heart disease. Its prevalence varies by patient’s underlying condition, occurring in up to 30–50% of advanced interstitial lung disease and a substantial proportion of patients with COPD. However, severe PH remains relatively uncommon. Despite the rareness of severe PH, the presence of PH has been consistently associated with functional limitation and increased mortality across chronic lung diseases [1,4,5,6].

### Pathophysiology of Group 3 PH

Group 3 PH arises from a heterogeneous group of chronic lung diseases and hypoxic conditions, including ILD, chronic COPD, obesity hypoventilation syndrome, and chronic exposure to high altitude. Across these conditions, loss of the pulmonary vascular bed, chronic hypoxic pulmonary vasoconstriction, endothelial dysfunction, inflammation, and pulmonary vascular remodeling contribute to increased PVR [7]. The relative contributions of these mechanisms vary according to the underlying lung disease, disease severity, and individual pulmonary vascular response.

**Figure 1:**
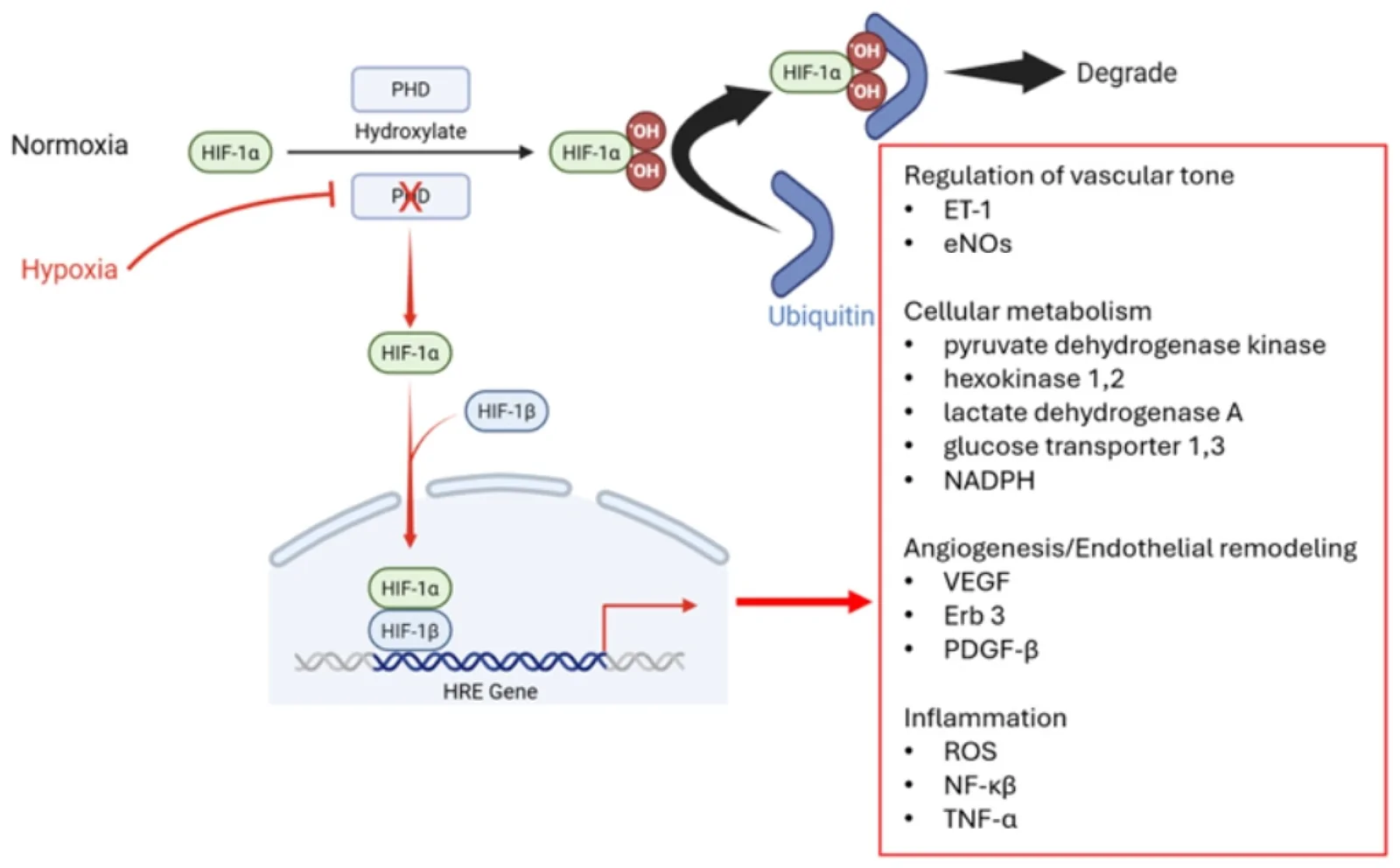
Summary of HIF-1α signaling. Under normoxic conditions, HIF-1α is hydroxylated, ubiquitinated, and targeted for proteasomal degradation. Under hypoxic conditions, reduced PHD activity stabilizes HIF-1α, allowing nuclear translocation and activation of transcriptional pathways involved in pulmonary vascular remodeling. HIF-1α signaling is implicated in both Group 3 PH and Group 1 PAH, highlighting a potential area of biological overlap between these disease groups.

Hypoxia-inducible factor (HIF) signaling is an important mediator of hypoxia-associated pulmonary vascular remodeling. During hypoxia, stabilization of HIF-1α and HIF-2α alters pathways involved in vascular tone, cellular metabolism, angiogenesis, inflammation, and vascular remodeling [8,9,10,11,12,13,14,15,16,17,18]. Together with oxidative stress and inflammatory signaling, these effects promote endothelial dysfunction, pulmonary arterial smooth muscle proliferation, medial hypertrophy, adventitial remodeling, and extracellular matrix deposition [11,12,13,14,15,16,17,18,19,20,21,22,23,24,25,26,27]. Cigarette smoke, bone morphogenetic protein (BMP)/transforming growth factor-β (TGF-β) signaling, and microRNAs may also contribute to pulmonary vascular remodeling. However, their roles as therapeutic targets in Group 3 PH are predominately supported by preclinical trials [17,28,29,30,31,32,33,34,35,36,37,38,39,40]. Importantly, HIF-1α signaling and several downstream remodeling pathways are also implicated in Group 1 pulmonary arterial hypertension (PAH), demonstrating that the biological mechanisms underlying classic Group 1 and Group 3 PH may overlap rather than being completely distinct [41,42] (Figure 1).

Accordingly, Group 3 PH encompasses a heterogeneous spectrum of pulmonary vascular phenotypes rather than a single pathophysiologic entity. In some patients, PH predominantly reflects advanced parenchymal lung disease, hypoxic vasoconstriction, and loss of the pulmonary vascular bed. In others, the severity of pulmonary vascular disease may appear disproportionate to the extent of parenchymal or ventilatory impairment, producing a pulmonary vascular phenotype with pathophysiologic features resembling those of Group 1 PAH. Although the initiating conditions differ, both phenotypes may involve endothelial dysfunction, imbalance between vasoconstrictor and vasodilator pathways, smooth muscle proliferation, and structural remodeling of the pulmonary circulation. These patients may be conceptualized as having an overlapping or mixed Group 1/Group 3 phenotype, although this remains a clinical construct without universally accepted diagnostic criteria [41]. Such patients represent plausible candidates for individualized evaluation at specialized PH centers, although greater responsiveness to PH-directed therapy has not been prospectively established. Conversely, when symptoms and hemodynamic impairment are driven predominantly by advanced parenchymal or ventilatory disease, the potential benefit of pulmonary vasodilation may be more limited and concerns regarding gas exchange may be greater. However, prospectively validated criteria for identifying treatment-responsive phenotypes remain lacking. Recognition of this heterogeneity is therefore essential for patient selection and allows for divergent results from clinical trials in Group 3 PH to be interpreted.

## 3. Right Ventricular Dysfunction and Failure in Pulmonary Hypertension

Chronic hypoxia, endothelial dysfunction, inflammation, oxidative stress, neurohormonal activation, myocardial ischemia, and altered cellular metabolism led to RV maladaptation. Increased RV wall tension raises myocardial oxygen demand, while systemic hypotension or markedly elevated RV pressures may reduce right coronary perfusion. These processes can promote fibrosis, loss of contractile reserve, and further deterioration of RV–PA coupling [43,44,45,46,47,48]. RV function is a major prognostic determinant in PH. RV dysfunction may be partially reversible when pulmonary vascular stress is effectively reduced, although the extent of reverse remodeling depends on the severity and duration of RV injury and remains incompletely defined in Group 3 PH [49,50]. This is important because RV dysfunction is a major prognostic determinant in PH.

PH increases right ventricular (RV) afterload through both resistive and pulsatile components of the pulmonary circulation. Pulmonary vascular resistance (PVR) reflects the resistive load, while reduced pulmonary arterial compliance and increased arterial elastance contribute to pulsatile loading [51]. Because the RV is a thin-walled chamber adapted to a low-pressure circulation, sustained pressure overload initially triggers compensatory increased contractility, concentric hypertrophy, and preservation of RV–pulmonary arterial (RV–PA) coupling, allowing maintenance of stroke volume and cardiac output.

As pulmonary vascular load progresses, RV contractile reserve may become insufficient, resulting in RV–PA uncoupling and maladaptive remodeling. This is characterized by RV dilation, increased wall stress, functional tricuspid regurgitation, myocardial fibrosis, and progressive systolic and diastolic dysfunction. RV enlargement and tricuspid regurgitation further increase volume overload, while interventricular septal flattening and leftward displacement impair left ventricular filling and reduce systemic cardiac output. The transition from compensated RV adaptation to RV–PA uncoupling and overt RV failure is therefore a major determinant of clinical deterioration and mortality in PH [43,44,45,52].

In Group 3 PH, RV dysfunction develops in the setting of chronic lung disease and reflects the combined effects of hypoxic pulmonary vasoconstriction, pulmonary vascular remodeling, and loss or compression of the pulmonary vascular bed. COPD-related hyperinflation may additionally impair venous return and cardiac filling, whereas restrictive mechanics in ILD can adversely affect cardiopulmonary interactions. Acute exacerbations, respiratory infections, worsening hypoxemia, or pulmonary embolism may abruptly increase pulmonary vascular load and precipitate acute-on-chronic RV failure [2,4,41,53,54,55,56]. Progressive RV dysfunction is associated with greater functional limitation, more frequent decompensation and hospitalization, and increased mortality. Prognosis depends not only on pulmonary arterial pressure or PVR, but critically on the ability of the RV to preserve cardiac output relative to the imposed pulmonary vascular load [44,45,57].

### Clinical Manifestations

Clinical manifestations arise from two principal consequences of RV failure: reduced forward cardiac output and systemic venous congestion. Early RV dysfunction primarily limits the ability to augment stroke volume during exercise, resulting in exertional dyspnea, fatigue, reduced exercise capacity, lightheadedness, or syncope. With progressive elevation of right-sided filling pressures, patients may develop jugular venous distention, peripheral edema, hepatic congestion, ascites, and cardiorenal dysfunction. Functional tricuspid regurgitation may further worsen congestion and RV volume overload.

Advanced RV failure is characterized by both congestion and inadequate systemic perfusion, with findings such as hypotension, oliguria, worsening renal function, cool extremities, altered mentation, and elevated lactate. In Group 3 PH, these manifestations may be amplified by impaired gas exchange and hypoxemia, and deterioration in RV function can produce symptoms disproportionate to changes in underlying lung function. Accordingly, worsening exercise tolerance, increasing oxygen requirements, progressive hypoxemia, recurrent edema, or new right-sided congestion should raise concern for clinically significant PH and RV dysfunction [2,4,45,53,54,55,56].

## 4. Diagnosis and Assessment of RV Dysfunction in Group 3 PH

Assessment of RV dysfunction in Group 3 PH requires integration of clinical evaluation, biomarkers, pulmonary and exercise testing, cardiac imaging, and invasive hemodynamics. No single non-invasive measurement adequately describes RV performance or RV–PA coupling, as summarized in Table 1. Echocardiography is the principal initial cardiac imaging modality, while right-heart catheterization (RHC) remains the gold standard for confirming PH and defining its hemodynamic phenotype [1,2].

Transthoracic echocardiography (TTE) is the principal non-invasive modality for evaluating patients with suspected pulmonary hypertension (PH), providing both an estimate of PH probability and an assessment of right ventricular (RV) remodeling and function. A comprehensive examination should assess peak tricuspid regurgitation velocity (TRV), RV and right atrial (RA) size, RV systolic function, tricuspid regurgitation, interventricular septal configuration, inferior vena cava characteristics, and other indicators of increased pulmonary vascular load. Peak TRV is commonly used for initial probability stratification: TRV ≤ 2.8 m/s generally indicates a low echocardiographic probability of PH, TRV 2.9–3.4 m/s an intermediate probability, and TRV > 3.4 m/s a high probability. However, TRV should not be interpreted in isolation because it may underestimate or overestimate pulmonary pressures and may be unobtainable, particularly in patients with chronic obstructive pulmonary disease (COPD), interstitial lung disease (ILD), hyperinflation, or poor acoustic windows. When TRV is ≤2.8 m/s or cannot be reliably measured, additional echocardiographic signs of pulmonary vascular disease and RV pressure overload should therefore be incorporated into the assessment rather than relying solely on estimated RV systolic pressure (RVSP) or pulmonary artery systolic pressure (PASP) [1].

Assessment of RV structure and systolic performance should similarly integrate multiple complementary parameters. Findings supportive of PH include RA area > 18 cm^2^, RV/LV basal diameter ratio > 1.0, interventricular septal flattening or LV eccentricity index > 1.1, RV outflow tract acceleration time < 105 ms and/or midsystolic notching, together with evidence of RV dilation or dysfunction. RV systolic function can be characterized using tricuspid annular plane systolic excursion (TAPSE), tissue Doppler-derived tricuspid annular systolic velocity (S′), RV fractional area change (RV-FAC), and RV free-wall longitudinal strain. A TAPSE < 17 mm or reduced RV-FAC supports impaired RV systolic function, while chamber enlargement and septal flattening indicate chronic RV pressure and volume overload. These structural and functional parameters are particularly valuable when reliable pulmonary pressure estimates cannot be obtained [1,58,59].

RV–pulmonary artery (RV–PA) coupling provides additional information regarding the ability of the RV to adapt to increased pulmonary vascular load. The TAPSE/sPAP ratio is the most widely used non-invasive echocardiographic surrogate, with values > 0.32 mm/mmHg suggesting relatively preserved or low-risk RV–PA coupling, 0.19–0.32 mm/mmHg indicating intermediate impairment, and <0.19 mm/mmHg indicating marked uncoupling and higher risk. Other proposed non-invasive measures include RV-FAC/PASP, RV free-wall longitudinal strain/PASP, and cardiac magnetic resonance-derived RV stroke volume/end-systolic volume ratios. These indices provide complementary information regarding RV adaptation but do not replace the invasive reference standard, the ratio of RV end-systolic elastance to pulmonary arterial elastance (Ees/Ea), derived from pressure–volume analysis [58,59].

In patients with Group 3 PH, particularly those with COPD or ILD, echocardiographic interpretation requires additional caution because hyperinflation, altered thoracic anatomy, and advanced parenchymal lung disease frequently limit acoustic windows and the accuracy of TRV-derived pressure estimates. Consequently, a normal or unmeasurable TRV does not reliably exclude PH. Diagnostic assessment should therefore integrate symptoms, pulmonary function testing, exercise testing, biomarkers, imaging, and echocardiographic evidence of RV remodeling or dysfunction, as no single non-invasive parameter is sufficiently reliable in this population.

When definitive hemodynamic characterization is required, right-heart catheterization remains the diagnostic standard for precapillary PH, defined by mean pulmonary artery pressure (mPAP) > 20 mmHg, pulmonary arterial wedge pressure (PAWP) ≤ 15 mmHg, and pulmonary vascular resistance (PVR) > 2 Wood units, with PVR calculated as (mPAP − PAWP)/cardiac output [1]. RHC directly measures right atrial pressure, pulmonary artery pressure, PAWP, cardiac output, cardiac index, and mixed venous oxygen saturation, thereby providing important information on both pulmonary vascular load and RV performance. Elevated right atrial pressure suggests systemic venous congestion, while reduced cardiac index, stroke volume, and mixed venous oxygen saturation indicate impaired forward flow and systemic oxygen delivery [1,44,45].

In Group 3 PH, RHC is particularly appropriate when severe PH is suspected, symptoms or functional limitation are disproportionate to the underlying lung disease, non-invasive findings are inconclusive or discordant, the PH phenotype is uncertain, or when hemodynamic confirmation will influence PH-specific therapy or lung-transplant evaluation. RHC should therefore be performed when its results are expected to meaningfully alter clinical management.

## 5. Clinical Management of Group 3 Pulmonary Hypertension

Management of Group 3 pulmonary hypertension (PH) begins with optimization of the underlying lung disease and correction of contributory factors rather than routine initiation of pulmonary arterial hypertension (PAH)-directed therapy. Treatment should be individualized according to the underlying pulmonary disorder, severity of PH, degree of right ventricular (RV) dysfunction, gas-exchange impairment, and overall clinical phenotype. Contemporary guidelines define severe PH associated with lung disease as a pulmonary vascular resistance (PVR) > 5 Wood units (WU), a threshold associated with worse prognosis and useful for identifying patients requiring specialized evaluation.

Patients with severe PH, significant RV dysfunction, diagnostic uncertainty, or consideration of PH-directed therapy should be referred to a specialized PH center [1].

Optimization of the underlying lung disease remains the cornerstone of management [1,7,60]. In chronic obstructive pulmonary disease (COPD), treatment should include guideline-directed inhaled therapy, smoking cessation, prevention and treatment of exacerbations, and other disease-specific interventions when appropriate. In interstitial lung disease (ILD), management should target the underlying disease, including antifibrotic or immunomodulatory therapy when indicated by the specific ILD phenotype [7,60].

Contributory conditions, including sleep-disordered breathing and alveolar hypoventilation, should also be identified and treated because they may coexist with parenchymal lung disease and independently contribute to hypoxemia and increased pulmonary vascular load [1,7,60]. Alternative or additional causes of PH, including left-sided heart disease and chronic thromboembolic disease, should also be evaluated when clinically suspected [1].

Correction of hypoxemia and optimization of functional status are important components of supportive management. Supplemental oxygen should be prescribed when clinically indicated, particularly in patients with resting or exertional hypoxemia, although evidence that long-term oxygen therapy modifies the natural history of PH outside COPD remains limited [1,7,60]. Noninvasive ventilation should be used when indicated for hypoventilation syndromes [1]. Pulmonary rehabilitation and structured exercise may improve functional capacity and quality of life and should be incorporated when tolerated [1,7,60].

PH-directed therapy should not be applied uniformly across Group 3 PH as treatment response and risk vary with the underlying lung disease and pulmonary vascular phenotype [1,41]. Pulmonary vasodilators may improve pulmonary hemodynamics but can have variable and potentially detrimental effects on gas exchange in patients with chronic lung disease, including worsening ventilation–perfusion mismatch [1]. Treatment decisions should therefore incorporate the severity of pulmonary vascular disease, extent of parenchymal lung disease, RV function, gas exchange, and evidence of a prominent pulmonary vascular phenotype [1,7,60].

Patient selection for targeted therapy is especially important given the heterogeneity of Group 3 PH. The population with the strongest evidence of benefit consists of patients with symptomatic, right-heart catheterization-confirmed PH associated with ILD (PH-ILD) who remain functionally limited despite optimization of the underlying ILD and supportive therapy. Evidence from the INCREASE trial supports consideration of inhaled treprostinil in appropriately selected patients within this population, as discussed in detail below [3]. Patients with severe PH-ILD, particularly those with PVR > 5 WU, RV dysfunction, or symptoms and hemodynamic impairment disproportionate to the extent of parenchymal lung disease, may also warrant individualized consideration of phosphodiesterase-5 inhibitor therapy at a specialized PH center, although supporting evidence remains limited [1,44]. In contrast, no COPD-PH phenotype has been prospectively demonstrated to derive consistent clinical benefit from targeted therapy, and the findings of the PERFECT trial do not support routine use of inhaled treprostinil in COPD-PH, as discussed later [61]. In patients with mild or nonsevere PH, advanced ventilatory limitation, marked hypoxemia, or substantial left-heart disease, the expected benefit of PH-directed therapy is uncertain, and the potential for adverse effects may be greater. Careful phenotyping—including confirmation of precapillary PH and exclusion of Group 1 PAH, chronic thromboembolic PH, and left-heart disease—is therefore essential before targeted therapy is initiated [1,7,41,60].

Referral to a specialized PH center should also facilitate consideration of advanced management strategies, including lung transplantation. PH complicating chronic lung disease is associated with worse functional status and prognosis, and eligible patients with progressive disease despite optimized therapy should be considered for timely transplant evaluation [1,7,60]. Recognition of severe pulmonary vascular disease and RV dysfunction may facilitate referral before advanced cardiopulmonary deterioration limits therapeutic options [1,7,60].

### 5.1. Pulmonary Hypertension Associated with Chronic Lung Disease (Group 3 PH)

Group 3 PH encompasses a heterogeneous spectrum of conditions driven by underlying parenchymal lung disease and chronic gas exchange abnormalities. Therapeutic approaches have been shaped by the unique vulnerability of these patients to V/Q mismatch, particularly in response to pulmonary vasodilators. Outcomes across different disease phenotypes, including COPD and ILD, highlight the importance of aligning pharmacologic interventions with underlying lung physiology [3,53,55,56,62].

### 5.2. COPD-Associated Pulmonary Hypertension: Limited Efficacy of Pulmonary Vasodilators

Pulmonary hypertension associated with COPD (COPD-PH) represents a distinct Group 3 phenotype characterized by chronic hypoxia, emphysematous vascular destruction, and marked ventilation heterogeneity. These factors contribute to an inherent susceptibility to V/Q mismatch with vasodilator therapy [3,53,55,56,63]. The prevalence of PH in COPD varies depending on disease severity, with estimates ranging from 10 to 35% in moderate-to-severe disease [53,55,56,64].

Systemic vasodilators, including ERAs and PDE5 inhibitors, have shown limited or inconsistent benefit [53,55,56,65,66,67]. However, the heterogeneity of COPD-PH raises the possibility that treatment response may differ according to the severity of pulmonary vascular disease relative to the degree of airflow obstruction and parenchymal lung disease.

### 5.3. Inhaled Prostacyclins in COPD-PH: Insights from PERFECT

The success of inhaled treprostinil in pulmonary hypertension associated with interstitial lung disease led to the hypothesis that inhaled prostacyclin therapy might also benefit patients with COPD-PH by producing pulmonary vasodilation while limiting disruption of V/Q matching. This rationale formed the basis for PERFECT, a multicenter, randomized, double-blind, placebo-controlled trial evaluating inhaled treprostinil in COPD-PH [3,61,68].

In the overall study population, inhaled treprostinil did not improve exercise capacity or symptoms and was associated with an unfavorable risk–benefit profile, including increased serious adverse events and mortality, resulting in early termination of the trial [3,61,68]. These findings demonstrate that the clinical benefits observed with inhaled treprostinil in PH-ILD cannot be generalized to an unselected COPD-PH population. Moreover, the mechanisms underlying the unfavorable outcomes remain uncertain and cannot be attributed solely to disruption of V/Q matching [61].

A subsequent post hoc analysis identified 7 of 66 patients exposed to inhaled treprostinil as potential responders. Potential responders had a baseline mean pulmonary artery pressure ≥ 40 mmHg and FEV_1_ ≥ 40% predicted, suggesting a phenotype characterized by severe pulmonary vascular involvement despite relatively preserved airflow. Conversely, all patients who died during the study had a baseline diffusing capacity of the lungs for carbon monoxide ≤ 25% predicted, potentially identifying a population with advanced parenchymal or pulmonary vascular disease that may be particularly vulnerable to adverse outcomes [68].

These post hoc findings were observational and exploratory, and the proposed thresholds require prospective validation. They therefore do not overcome the unfavorable overall findings of PERFECT or support routine use of inhaled treprostinil in COPD-PH. Nevertheless, they support further prospective investigation into whether a carefully selected pulmonary vascular-predominant phenotype—characterized by severe PH disproportionate to the degree of airflow obstruction—might respond differently to PH-directed therapy. Future COPD-PH trials should incorporate hemodynamic severity, spirometry, diffusing capacity, imaging morphology, and RV phenotype into patient selection [68].

### 5.4. Pulmonary Hypertension Associated with Interstitial Lung Disease

PH-ILD includes a heterogeneous group of fibrotic lung disorders, such as idiopathic pulmonary fibrosis (IPF) and other idiopathic interstitial pneumonias (IIP). PH-ILD frequently features substantial diffusion impairment and pulmonary vascular remodeling, although the extent and relative contribution of pulmonary vascular involvement vary considerably across both ILD and COPD phenotypes [4,5,53,55,56].

### 5.5. Systemic Pulmonary Vasodilators in ILD: Predominantly Negative or Inconclusive Trial Evidence

Endothelin receptor antagonists have consistently failed to demonstrate benefit when evaluated as treatments for IPF or other fibrotic ILDs; however, these trials generally did not specifically enroll patients with hemodynamically confirmed PH-ILD. In ARTEMIS-IPF, ambrisentan was associated with increased disease progression and respiratory hospitalizations, leading to early termination [29,69]. Although approximately 10% of participants had PH reported at baseline, the trial was neither designed nor adequately powered to assess efficacy specifically in IPF-PH. Accordingly, its negative findings should be extrapolated cautiously to carefully phenotyped patients with IPF-PH or other forms of PH-ILD, although the observed safety signal and absence of supportive randomized evidence argue against routine use. Similarly, the BUILD-1 and BUILD-3 trials evaluating bosentan, as well as the MUSIC trial evaluating macitentan, failed to improve exercise capacity or slow disease progression [53,55,56,66,67,70]. Evidence from studies specifically targeting PH-ILD has likewise failed to establish a therapeutic role for endothelin receptor antagonists. In a randomized, right heart catheterization-confirmed trial, bosentan failed to improve PVR, exercise capacity, or clinical outcomes [71].

In contrast to the consistently negative findings with endothelin receptor antagonists, phosphodiesterase-5 inhibitors have shown mixed results in IPF. In the STEP-IPF trial, sildenafil did not meet the primary endpoint of improving exercise capacity in patients with advanced IPF. However, modest improvements in dyspnea, quality of life, and oxygenation were observed in the overall study population [65]. Importantly, a prespecified echocardiographic substudy found that sildenafil was associated with better preservation of exercise capacity and improved quality of life among patients with right ventricular systolic dysfunction [65,72]. Because RV dysfunction may reflect a more prominent pulmonary vascular phenotype within chronic lung disease-associated PH, these findings suggest that selected patients with disproportionate pulmonary vascular involvement may be more responsive to PH-directed therapy [41]. However, STEP-IPF did not require right-heart catheterization-confirmed PH, and the small subgroup analysis should be considered hypothesis-generating rather than evidence supporting routine sildenafil use in unselected patients with IPF or PH-ILD [65,72]. The INSTAGE trial evaluating sildenafil added to nintedanib in patients with advanced IPF also failed to demonstrate significant improvements in quality of life or lung function, including in subgroup analyses based on the presence of echocardiographic right-heart dysfunction [53,55,56,70].

### 5.6. RISE-IIP: An Unfavorable Safety Signal with Riociguat in PH-IIP

The RISE-IIP trial represents a pivotal study in PH-ILD. This randomized, double-blinded, placebo-controlled trial evaluated riociguat in patients with IIPs and RHC-confirmed [53,55,73]. Despite RHC confirmation and a rigorous randomized design, the trial was terminated early due to increased serious adverse events and mortality in the treatment group, without improvement in exercise capacity [73]. These findings established an unfavorable risk–benefit profile for riociguat in PH associated with idiopathic interstitial pneumonia. It raised concerns that systemic pulmonary vasodilation may worsen gas exchange by impairing hypoxic pulmonary vasoconstriction and increasing V/Q mismatch and intrapulmonary shunting [1,2,73].

However, a subsequent post hoc imaging analysis suggested that lung morphology may have influenced the observed outcomes. Among participants with available high-resolution computed tomography (HRCT) imaging, mortality was higher in patients with combined pulmonary fibrosis and emphysema than in those without emphysema (29% vs. 13%), and a high parenchymal disease burden or emphysema exceeding fibrosis may have predisposed patients to poor outcomes [74]. Notably, HRCT imaging was available for only 65 of the 147 participants, and no relationship was demonstrated among combined pulmonary fibrosis and emphysema score, survival status, and treatment assignment; therefore, these findings remain exploratory and hypothesis-generating [74]. Thus, RISE-IIP supports avoiding use of riociguat in PH associated with idiopathic interstitial pneumonia but should not be interpreted as establishing a uniform negative safety signal for all systemic pulmonary vasodilators across the heterogeneous spectrum of PH-ILD [73,74]. These findings also emphasize the importance of centrally adjudicated imaging and morphologic phenotyping in future Group 3 PH trials [74].

### 5.7. Transition to Lung-Selective Therapy

Despite these negative and safety-limiting findings from systemic vasodilator trials, they collectively contributed to a critical refinement of the understanding of the pathophysiology of PH-ILD [2,71,73,75]. Rather than indicating a lack of therapeutic targets, these studies highlighted the clinical limitations and potential safety concerns associated with nonselective pulmonary vasodilation in fibrotic lung disease. Worsening V/Q matching remained one biologically plausible concern, although it was not directly established as the mechanism underlying the unfavorable outcomes [2]. This evolving insight prompted a shift in therapeutic strategy toward interventions that preferentially target well-ventilated lung regions while preserving gas exchange [75].

Within this context, lung-selective delivery of vasodilators emerged as a rational next step, ultimately culminating in the INCREASE trial, which evaluated whether inhaled treprostinil could improve clinical outcomes in PH-ILD while limiting adverse effects on oxygenation [2,3,75].

### 5.8. Selected Signals in Severe PH-ILD: Evidence from Highly Enriched Populations

In contrast to broadly negative randomized trials, smaller studies in highly selected patients with severe PH and RV dysfunction have suggested potential benefit from prostacyclin therapy. In a pilot study of intravenous treprostinil in advanced fibrotic ILD with severe PH, treatment was associated with improvements in hemodynamics, RV function, and exercise capacity without worsening oxygenation [63].

However, the study was limited by its small sample size, open-label nonrandomized design, and highly selected transplant-referral population; its findings should therefore be considered hypothesis-generating [1,2,63]. Nevertheless, these observations suggest that therapeutic response may depend not only on the route of drug delivery but also on the severity of pulmonary vascular disease and the presence of RV dysfunction.

### 5.9. Inhaled Lung-Selective Vasodilators: Redefining Treatment in PH-ILD

The INCREASE trial marked a major paradigm shift in the treatment of PH-ILD. Inhaled administration was hypothesized to preferentially deliver treprostinil to better-ventilated lung regions, thereby reducing the risk of unfavorable perfusion redistribution compared with systemic vasodilation. However, INCREASE did not directly measure regional aerosol deposition, redistribution of pulmonary perfusion, or V/Q matching; this mechanism therefore remains a biologically plausible mechanism rather than a definitively established explanation for the trial’s success [3,53,55,56].

In this randomized, placebo-controlled trial of 326 patients with RHC-confirmed PH-ILD, inhaled treprostinil improved six-minute walk distance, reduced NT-proBNP, and decreased clinical-worsening events, without a significant treatment-related deterioration in oxygenation [3]. These findings led to inhaled treprostinil becoming the first approved therapy for PH-ILD [1,3,69]. Importantly, prespecified subgroup analysis suggested heterogeneity by baseline pulmonary vascular burden: improvement in six-minute walk distance was observed predominantly among patients with PVR of at least 4 WU, whereas patients with PVR below 4 WU did not demonstrate benefit and showed a numerical trend toward worsening six-minute walk distance [3,76]. A subsequent systematic assessment identified a statistically significant interaction between baseline PVR and treatment response. Still, it characterized the finding as having only moderate credibility and therefore as exploratory and hypothesis-generating rather than sufficient to direct individual treatment decisions [76].

The relationship between baseline PVR and exercise response challenges the assumption that preferential delivery to ventilated lung regions alone explains the efficacy of inhaled treprostinil. Patients with higher PVR may have a more prominent and potentially reversible pulmonary vascular phenotype, with pulmonary vascular disease and increased RV afterload contributing substantially to exercise limitation. Conversely, patients with lower PVR may have exercise limitations driven predominantly by parenchymal restriction, impaired diffusion, hypoxemia, or deconditioning, and may therefore derive less benefit from pulmonary vasodilation [1,2,3,76]. In such patients, pulmonary vasodilation may provide insufficient hemodynamic benefit relative to its adverse effects; whether unfavorable perfusion redistribution contributes remains uncertain [1,2]. Nevertheless, the lower-PVR subgroup was relatively small, and INCREASE was not powered to establish efficacy or harm within individual PVR strata; consequently, the observed numerical trend should not be interpreted as definitive evidence of treatment-related harm [3,76].

Furthermore, a subsequent post hoc analysis found that patients with less severe hemodynamic impairment who received inhaled treprostinil experienced fewer exacerbations and clinical worsening events, and reductions in NT-proBNP [77]. In the open-label extension, earlier treatment was also associated with possible longer-term benefits in exercise capacity and clinical outcomes, although these subgroup analyses were underpowered and require prospective confirmation [77]. Thus, the absence of short-term improvement in six-minute walk distance does not necessarily indicate an absence of benefit across other outcomes. Inhaled administration may help preserve gas exchange, while the magnitude and type of clinical benefit may depend on the underlying pulmonary vascular phenotype and the outcome evaluated [1,76,77].

The unfavorable findings with inhaled treprostinil in PH-COPD in the PERFECT trial further challenge the assumption that inhaled delivery uniformly prevents clinically important V/Q mismatch or ensures efficacy and safety across Group 3 PH. PERFECT was terminated early because of increased serious adverse events, a possible mortality signal, and no improvement in exercise capacity [61]. However, regional perfusion and V/Q matching were not directly measured, and worsening V/Q mismatch was not established as the cause of harm. The contrasting outcomes of INCREASE and PERFECT suggest that therapeutic response reflects an interaction among the underlying parenchymal disease, pulmonary vascular burden, RV function, regional aerosol deposition, and gas-exchange reserve rather than the inhaled route of administration alone [1,2,3,61].

Additional evidence regarding inhaled pulmonary vasodilation comes from inhaled nitric oxide studies, which reported improvements in physical activity or exercise physiology without significant deterioration in oxygenation [78,79]. However, preservation of oxygenation should not be interpreted as direct proof of preferential regional vasodilation or improved V/Q matching [1,2,78].

### 5.10. Key Interpretation

Across Group 3 PH, therapeutic response depends on the interaction among the underlying lung disease phenotype, pulmonary vascular burden, RV function, gas-exchange reserve, pharmacologic mechanism, and route of administration. Trials of systemic pulmonary vasodilators have generally demonstrated limited efficacy or unfavorable safety findings, although the interpretation depends on the study population and whether PH was hemodynamically confirmed. The contrasting outcomes of INCREASE and PERFECT are particularly informative because both evaluated inhaled treprostinil: INCREASE demonstrated clinical benefit in PH-ILD, whereas PERFECT showed an unfavorable overall risk–benefit profile in COPD-PH. This contrast indicates that inhaled delivery alone does not ensure efficacy or safety and that preferential vasodilation of ventilated lung regions remains a proposed rather than directly established explanation for benefit. Exploratory subgroup findings from both trials further suggest that the magnitude of pulmonary vascular involvement relative to parenchymal lung disease may influence treatment response. Collectively, the evidence supports careful phenotyping rather than uniform application of PH-directed therapy across Group 3 PH populations [3,53,55,56,61,62,68,77].

In clinical practice, patients most appropriate for consideration of PH-directed therapy are those with RHC-confirmed precapillary PH in whom pulmonary vascular disease appears to contribute substantially to symptoms, exercise limitation, or RV dysfunction despite optimization of the underlying lung disease and correction of hypoxemia [1,2,60]. A pulmonary vascular-predominant phenotype may be suggested by severe PH, currently defined by PVR greater than 5 WU; symptoms or circulatory limitation disproportionate to ventilatory impairment; markedly reduced DLCO; RV enlargement or dysfunction; elevated BNP or NT-proBNP; and relatively modest parenchymal abnormalities despite substantial hemodynamic impairment [1,2,41]. No single feature or hemodynamic threshold establishes treatment candidacy; identification requires integration of clinical findings, pulmonary function testing, high-resolution CT, echocardiography, biomarkers, exercise assessment, and invasive hemodynamics, together with exclusion of left-heart disease, chronic thromboembolic disease, and coexisting Group 1 PAH [1,41,60,80]. These findings should prompt evaluation at an expert PH center rather than automatic initiation of therapy, because treatment remains disease- and drug-specific [1,60].

### 5.11. Future Directions: Lung-Selective and Phenotype-Directed Therapy

Future research should prioritize precise phenotyping rather than treating Group 3 PH as a single clinical entity. PH-ILD and PH-COPD should be investigated separately, with further stratification by the underlying lung disease subtype, extent of parenchymal involvement, comorbidities, gas-exchange impairment, and severity of pulmonary vascular disease. Particular attention should be given to identifying patients whose pulmonary vascular disease is disproportionately severe relative to their underlying parenchymal or ventilatory impairment. Integration of invasive hemodynamics, quantitative imaging, pulmonary function testing, exercise physiology, circulating biomarkers, and right ventricular assessment may help define clinically meaningful endotypes, facilitate earlier detection and risk stratification, and predict disease progression and treatment response [13,41,78,81]. Longitudinal studies are also needed to determine how these measures evolve and to identify the optimal timing for referral to a specialized PH center, right-heart catheterization, and therapeutic intervention.

Therapeutic development should focus on disease-modifying interventions that attenuate pulmonary vascular remodeling without worsening oxygenation or ventilation–perfusion matching. Lung-selective delivery remains promising; however, the divergent outcomes of INCREASE in PH-ILD and PERFECT in PH-COPD demonstrate that inhaled administration alone does not ensure efficacy or safety and suggest that the underlying lung disease phenotype significantly influences treatment response [3,61,75]. Future studies should investigate the mechanisms responsible for beneficial and adverse responses to pulmonary vasodilator therapy and determine whether clinical, imaging, physiologic, or molecular markers can identify potential responders before treatment. Further investigation is also needed to determine whether pathways implicated in experimental Group 3 PH—including hypoxia-inducible factor signaling, endothelial dysfunction, inflammation, altered cellular metabolism, and microRNA regulation—can be translated into therapies that reduce vascular remodeling without compromising gas exchange [2].

Lung-selective treatment should extend beyond the route of drug administration to encompass molecular and cellular strategies targeting the diseased pulmonary circulation. Further investigation is needed to determine whether pathways implicated in experimental Group 3 PH—including hypoxia-inducible factor signaling, endothelial dysfunction, inflammation, altered cellular metabolism, and microRNA regulation—can be translated into therapies that limit pulmonary vascular remodeling without compromising gas exchange [2]. Because these pathways may also participate in adaptive responses to hypoxia and interact with inflammatory and fibrotic processes in the lung, therapeutic modulation will require careful validation in disease-specific models before clinical application.

Because right ventricular dysfunction is a major determinant of symptoms and prognosis, future investigation should extend beyond the pulmonary vasculature to characterize right ventricular–pulmonary arterial coupling, identify early markers of right ventricular maladaptation, and evaluate interventions that preserve right ventricular function. Future trials should use disease-specific eligibility criteria and incorporate clinically meaningful outcomes beyond short-term changes in 6 min walk distance, including clinical worsening, hospitalization, oxygen requirements, right ventricular function, health-related quality of life, transplant-free survival, and overall survival [60,82]. Standardized definitions of disease severity and treatment response, together with multicenter registries and longer-term pragmatic studies, will be important for translating clinical-trial findings into routine practice.

## 6. Conclusions

Group 3 pulmonary hypertension reflects a complex interplay among pulmonary vascular remodeling, underlying lung disease, chronic hypoxia, inflammation, and right ventricular maladaptation. Despite advances in understanding these mechanisms, effective therapies remain limited. Clinical trials of systemic pulmonary vasodilators have generally demonstrated inconsistent benefit and, in some populations, unfavorable safety outcomes. Although worsening ventilation–perfusion matching and gas exchange remain biologically plausible concerns, these mechanisms were not directly established as causes of harm in the major randomized trials. In contrast, the efficacy of inhaled treprostinil in PH-ILD represents an important advance toward lung-selective therapy, whereas the adverse findings of RISE-IIP and the unfavorable risk–benefit profile observed in PERFECT emphasize the heterogeneity of Group 3 PH and the need for disease-specific treatment strategies [1,3,4,5,61,73,75].

Optimization of the underlying lung disease, correction of hypoxemia, supportive management of RV dysfunction, and appropriate referral to specialized PH centers remain the foundations of care. Ultimately, progress will depend on integrating mechanistic understanding with precise clinical phenotyping to match carefully selected patients to therapies that address pulmonary vascular disease while preserving gas exchange.

## Figures and Tables

**Table 1:** Clinical, physiologic, imaging, and hemodynamic indicators of Group 3 PH.

Diagnostic Domain	Key Findings Suggestive of PH	Clinical Significance
**Pulmonary Function Tests (PFTs)**	DLCO < 40% predicted (especially <30–32%); FVC/DLCO > 1.4; TLC/DLCO > 1.67; progressive DLCO decline despite stable FVC	Reduced gas transfer is a strong indicator of pulmonary vascular involvement and worse prognosis
**Exercise Testing**	Reduced 6 min walk distance (6MWD), greater exertional desaturation, heart rate recovery < 13 bpm at 1 min	Suggests impaired cardiopulmonary reserve and possible PH
**Cardiopulmonary Exercise Testing (CPET)**	Reduced oxygen pulse, lower peak VO_2_, increased ventilatory inefficiency (VE/VCO_2_)	Demonstrates circulatory limitation consistent with PH
**Biomarkers**	Elevated BNP or NT-proBNP; BNP ≥ 20 pmol/L in ILD associated with increased mortality	Reflects right ventricular (RV) strain and disease severity
**Electrocardiography (ECG)**	Right axis deviation, P pulmonale, RV strain pattern	Supportive of PH or RV modeling but lacks sensitivity and specificity
**Computed Tomography (CT)**	Main pulmonary artery diameter > 29 mm; PA:Aorta ratio > 1; RV enlargement; increased RV/LV ratio	Suggestive of PH and useful for assessing underlying lung disease

## Data Availability

No new data were created or analyzed in this study. Data sharing is not applicable to this article.
